# Supplementary material for: Modelling neural coding in the auditory midbrain with high resolution and accuracy
Source: Nat Mach Intell. 2025 Sep 18;7(9):1478–93. doi: 10.1038/s42256-025-01104-9 (PMC12460167; doi:10.1038/s42256-025-01104-9)
Supplement: Supplementary file 1 — Supplementary Figs. 1–5. [file 42256_2025_1104_MOESM1_ESM.pdf]

nature machine intelligence

Supplementary information https://doi.org/10.1038/s42256-025-01104-9

Modelling neural coding in the auditory midbrain with high resolution and accuracy

In the format provided by the authors and unedited

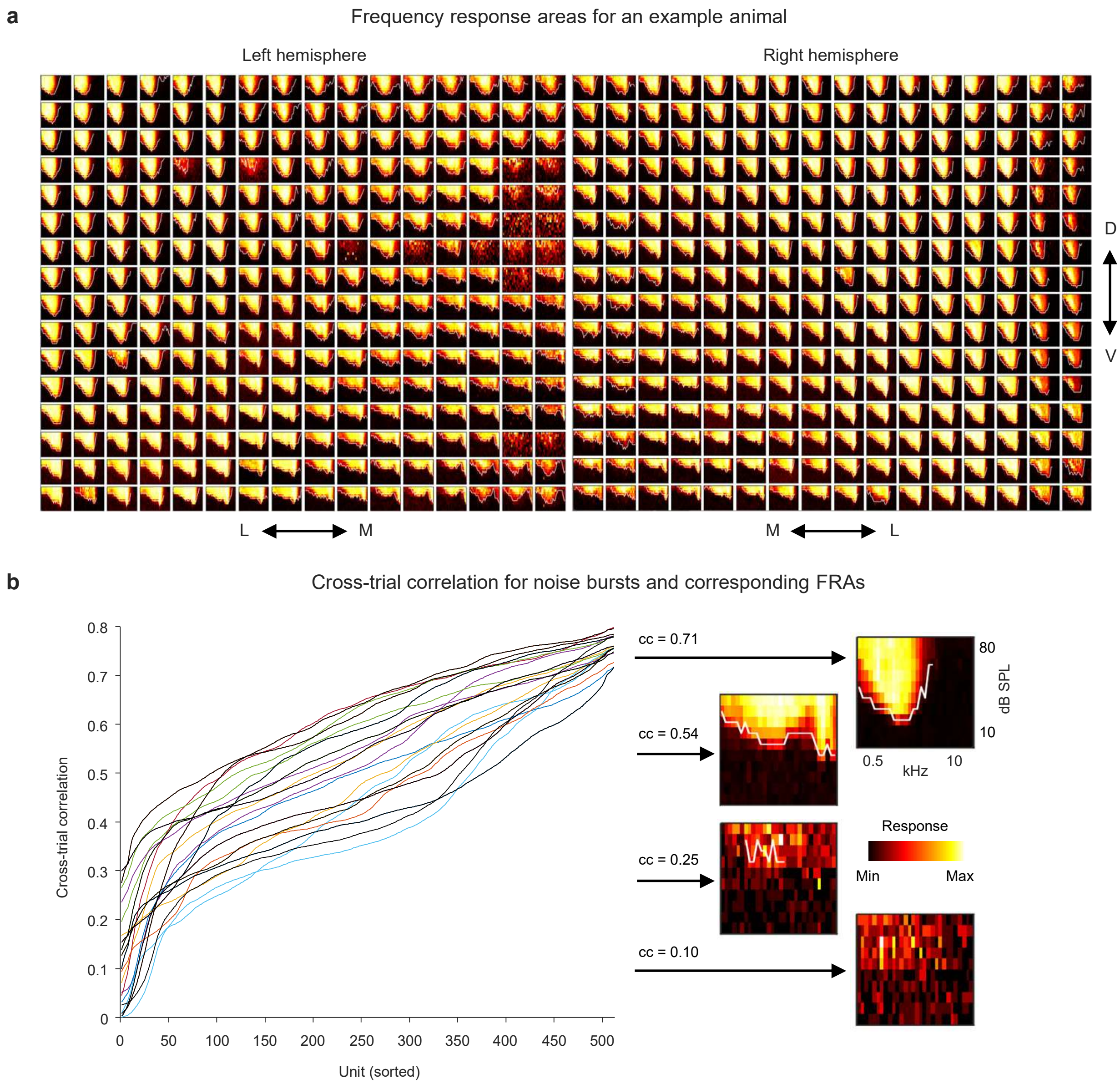

Supplementary Fig. 1. Identification of "good" recording sites. We have described our approach to targeting the central nucleus of the inferior colliculus in a previous paper (see Fig. S1 in [22]). We used the same approach for the recordings that we used in the development of ICNet. Our electrodes are designed to span the mediolateral extent of the central nucleus. Panel **a** shows frequency response areas (FRAs) estimated from MUA recorded on 512 recording sites for one example animal (each group of two columns represents 1 of 8 shanks on each electrode; the arrows indicate medial, lateral, dorsal, and ventral). Aside from a few scattered damaged sites, most of the sites exhibit the clear "V-shaped" FRAs associated with the central nucleus. Only some of the most medial sites on the left electrode and the most lateral sites on the right electrode exhibit FRAs that are not clear and V-shaped. As an objective quantitative criterion to identify (undamaged) sites that are likely to lie within the central nucleus, we calculated the cross-trial correlation of responses to broadband noise bursts and then sorted the sites by this cross-trial correlation value in increasing order. The results after sorting are shown in panel **b** for 20 normal hearing animals, with the 9 animals used to train ICNet shown in black and other animals shown in color. Sites with high cross-trial correlation values also have clear V-shaped FRAs, whereas sites with low cross-trial correlation values do not. We included all sites in model training but excluded sites with cross-trial correlation < 0.2 for evaluation.

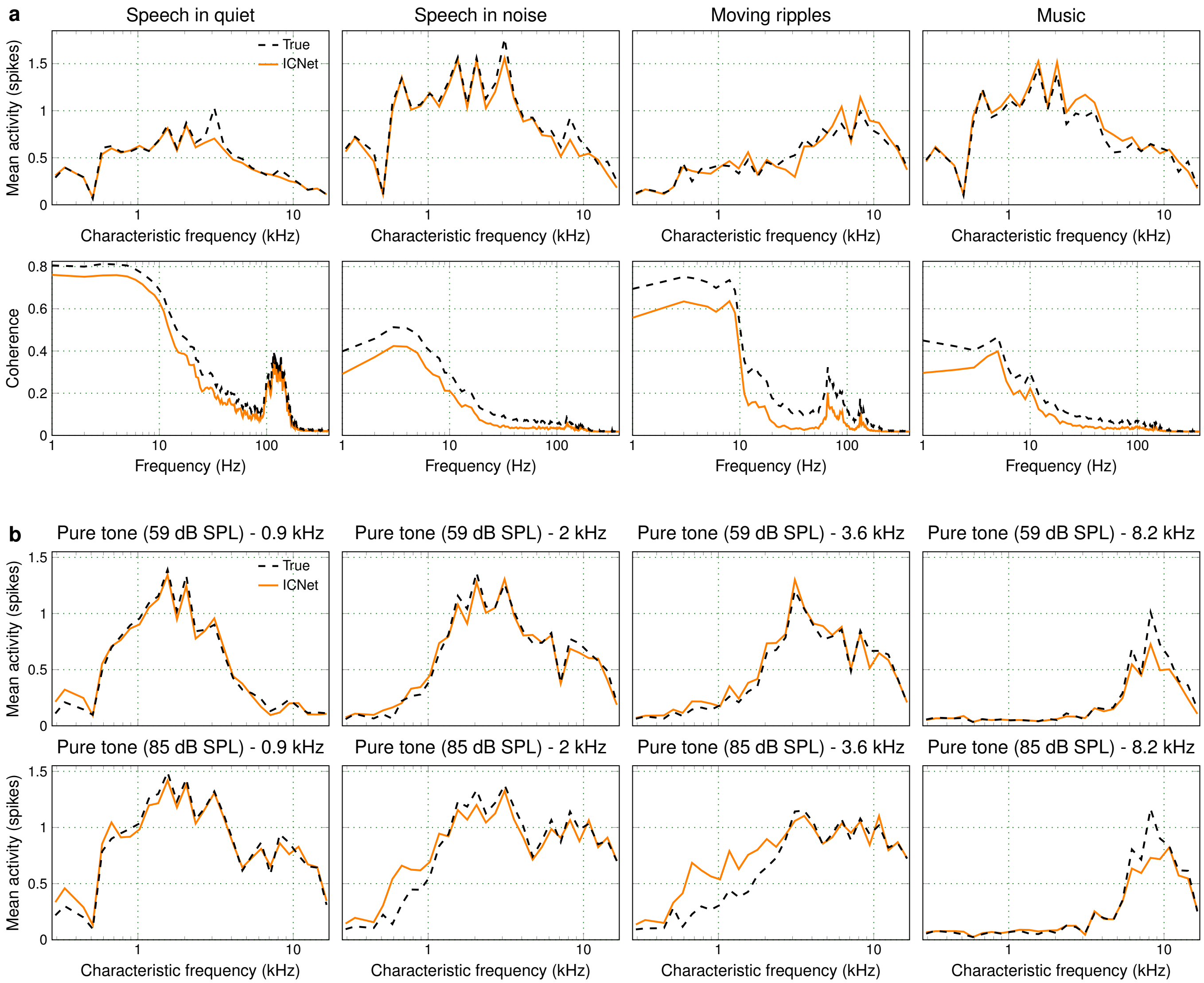

Supplementary Fig. 2. Assessment of systematic errors. The top row of panel **a** shows the overall activity (mean of counts across time bins) of 3476 units from 7 animals along with that predicted by ICNet in response to our 4 primary evaluation sounds. The results are grouped and sorted based on the CF of each unit. The bottom row of panel **a** shows the average coherence spectrum (across units) between either the recorded responses across trials or the recorded and predicted responses for the same 4 sounds. Overall, we found little evidence of systematic errors in ICNet's predictions of responses to complex sounds. Panel **b** shows the overall activity of 4446 units from 9 animals along with that predicted by ICNet in response to pure tones at 4 frequencies and 2 intensities. When presented with pure tones of 2 and 3.6 kHz at 85 dB SPL, ICNet overpredicted the overall activity for units with CFs below the tone frequency (frequencies between 0.5 and 2 kHz). This was not evident for the tones presented at 59 dB SPL.

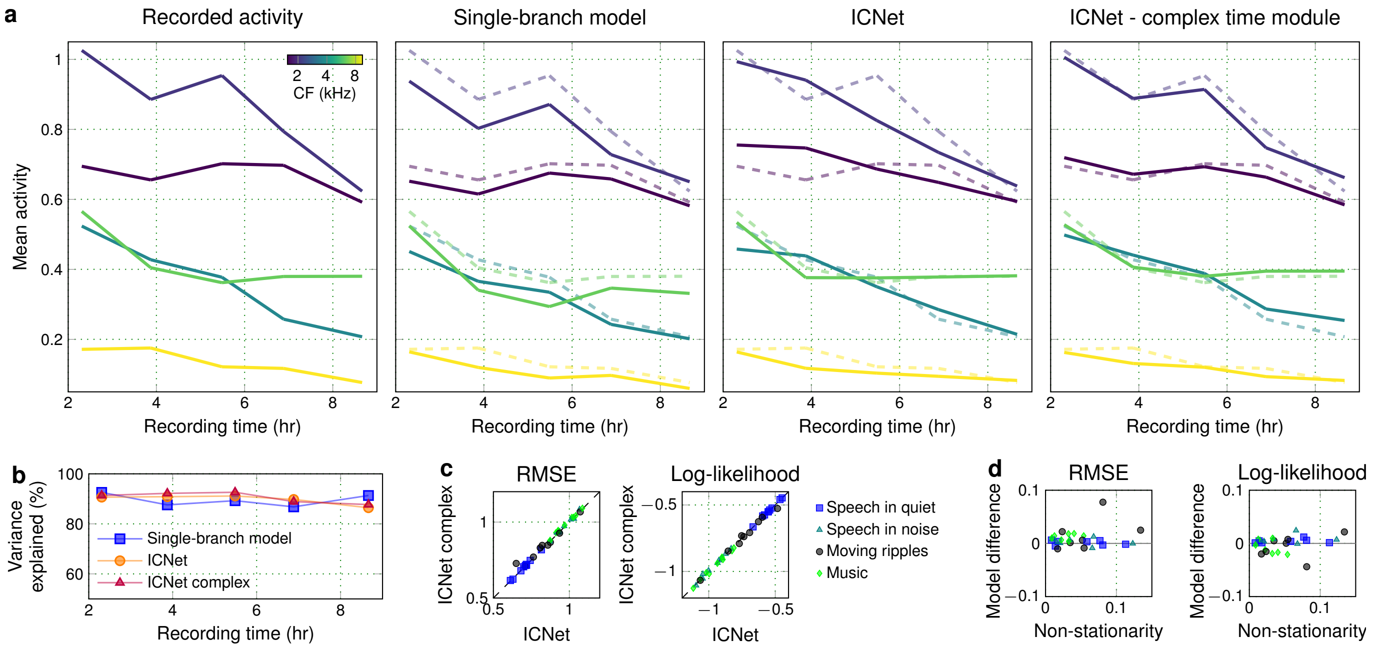

Supplementary Fig. 3. Accounting for non-stationarity in neural recordings. The long duration of our recordings had varying effects across units, with non-uniform and, in some cases, non-monotonic changes in overall activity for non-stationary recordings. We tested time modules with varying capacity in the ICNet architecture, but found that the additional capacity required to capture the full complexity of non-stationarity did not significantly improve overall performance. The first plot in panel **a** shows the mean activity of 5 example units (from the non-stationary recording of Fig. 2b) in response to 5 presentations of the same speech sound at different times, with the units chosen to reflect the range of non-uniform and non-monotonic effects of non-stationarity in our recordings. The second plot in panel **a** compares the recorded activity to the predictions of the single-branch time-variant architecture (Fig. 2), which was able to capture the full effects of the non-stationarity. The third plot in panel **a** shows the predictions of the final ICNet, which uses a time module with limited capacity and is only able to capture monotonic trends. The fourth plot in panel **a** shows the predictions of ICNet with a more complex time module (see Methods), which was again able to capture the full effects of the non-stationarity. We compared the performance of the 3 model architectures (single-branch, final ICNet, and ICNet with complex time module) with respect to their overall predictive power as in Fig. 2. Panel **b** shows the median predictive power of the 3 model architectures across all units at 5 different times during the non-stationary recording of Fig. 2b. Panel **c** shows a performance comparison across 9 animals and 4 sounds between the two variations of ICNet. Panel **d** shows the performance difference between the two varations of ICNet (ICNet with complex time module − final ICNet) as a function of recording non-stationarity across 7 animals and 4 sounds. Because there was little evidence of performance benefit for the more complex time module, we opted for the simpler time module for the final ICNet.

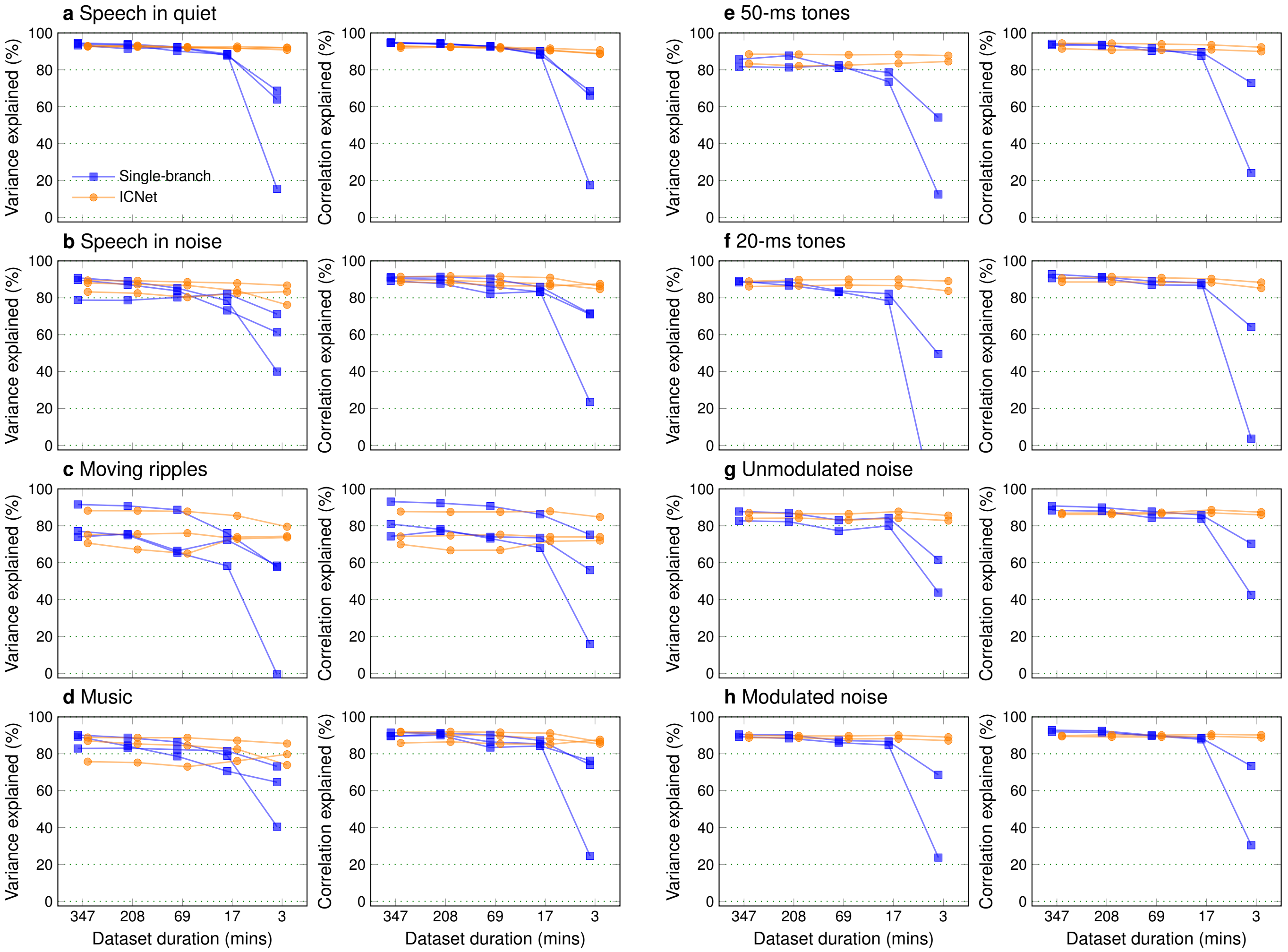

Supplementary Fig. 4. Generality of ICNet across new sounds and animals. We used neural recordings from new animals (unseen during ICNet training) to train single-branch and ICNet-based models. We trained the single-branch models from scratch (learning all parameters), and trained the ICNet-based models by freezing the ICNet encoder and learning only a new decoder. We trained both model variants using the abridged training dataset (347 minutes; see Methods) and sub-portions of it which were generated by keeping 60%, 20%, 5% and 1% of the dataset, resulting in training datasets with durations of 208, 69, 17 and 3 minutes, respectively. We then assessed the performance of all trained models across different sounds (unseen during training) as a function of the amount of data used for training. Panels **a**-**d** show model performance across 3 new animals on the 4 sounds that were used for our primary ICNet evaluation (Fig. 4). Panels **e**-**h** show model performance across 2 new animals on the sounds that were used to assess frequency tuning, forward masking and amplitude-modulation tuning (Fig. 5; sounds from the neurophysiological evaluation dataset for which we recorded responses to repeated trials). The results show that (1) ICNet performed as well as fully-trained single-branch models and (2) that this level of performance could be achieved with as little as 3 minutes of data to retrain the decoder.

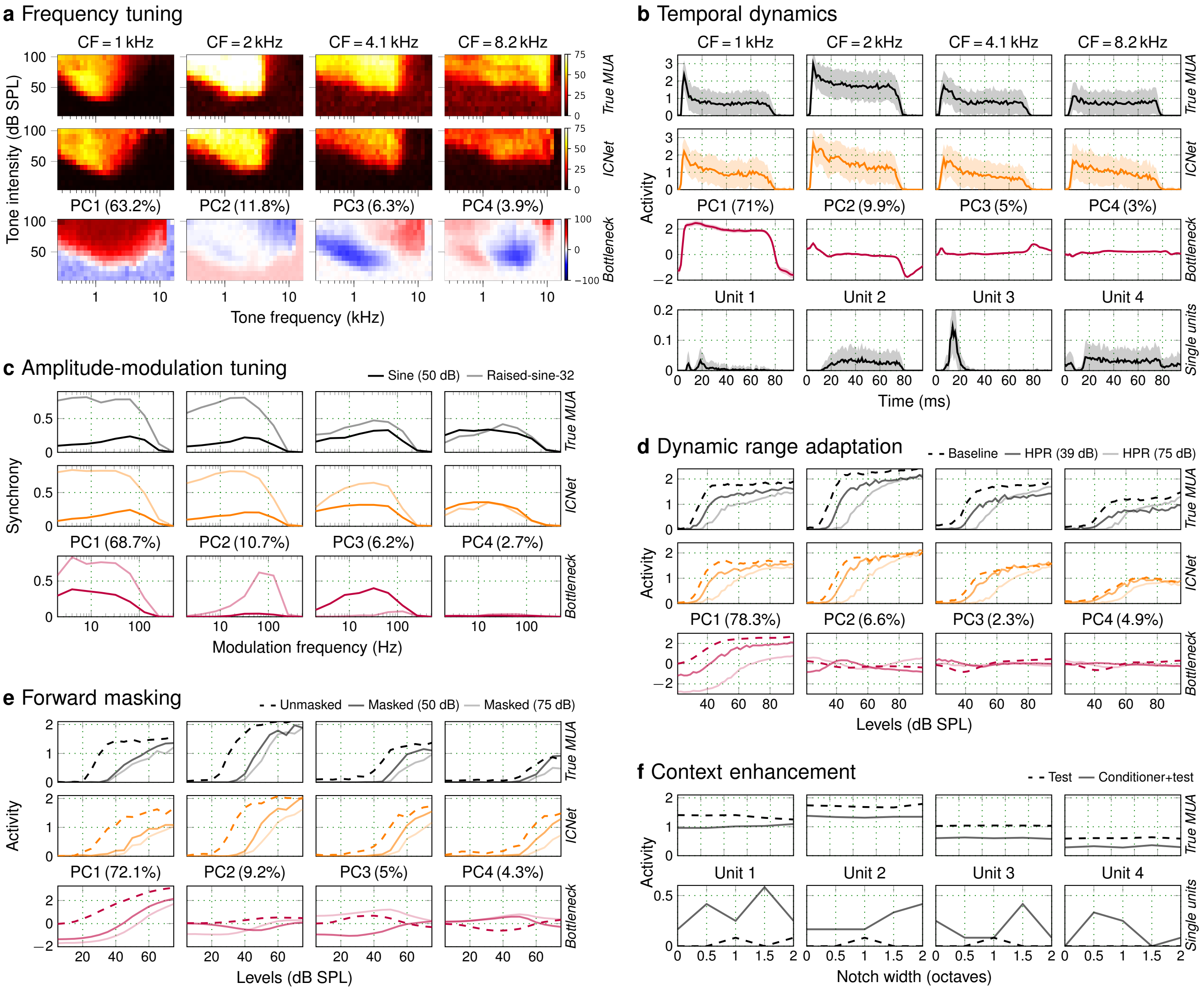

Supplementary Fig. 5. ICNet captures fundamental neurophysiological phenomena. The results of Fig. 5, repeated for another animal. **a.** Recorded (top row) and predicted (middle row) frequency response areas (FRAs) for 4 example neural units with a range of CFs. Each panel shows the overall activity to pure tones of varying intensity and frequency. The FRAs for the top 4 principal components (PCs) of the ICNet bottleneck responses are also shown (bottom row), with the numbers in the parentheses indicating the percentage of the total variance in the bottleneck responses captured by each component. **b.** Temporal dynamics in response to a 2-kHz pure tone stimulus presented at 85 dB SPL. The lines and shaded regions indicate the mean and standard deviation across 128 trials. Responses from 4 example single units are also shown (bottom row). **c.** Synchrony to the envelope of an amplitude-modulated narrowband noise (centered at 2 kHz) with two different envelopes. **d.** Rate-intensity functions for broadband noise. The noise intensity was drawn from distributions that were either uniform (baseline) or contained high-probability regions (HPRs) centered at one of two intensities. **e.** Rate-intensity functions for a 2-kHz pure tone that was preceded either by silence (unmasked) or by a masker tone presented at one of two intensities. **f.** Overall activity in response to a multi-tonal complex with a spectral notch centered at 1 kHz. A 1-kHz tone was presented either concurrently with the multi-tonal complex (test) or after some delay (conditioner+test). The overall activity of 4 example single units is also shown (bottom row).
